# High-Throughput Microplate-Based Assay to Monitor Plasma Membrane Wounding and Repair

**DOI:** 10.3389/fcimb.2017.00305

**Published:** 2017-07-14

**Authors:** Sarika Pathak-Sharma, Xiaoli Zhang, Jonathan G. T. Lam, Noah Weisleder, Stephanie M. Seveau

**Affiliations:** ^1^Department of Microbial Infection and Immunity, The Ohio State University Medical Center Columbus, OH, United States; ^2^Department of Biomedical Informatics, Center for Biostatistics, The Ohio State University Medical Center Columbus, OH, United States; ^3^Department of Microbiology, The Ohio State University Columbus, OH, United States; ^4^Department of Physiology and Cell Biology, The Ohio State University Medical Center, Davis Heart and Lung Research Institute Columbus, OH, United States; ^5^Center for Microbial Infection Biology, The Ohio State University Medical Center Columbus, OH, United States

**Keywords:** plasma membrane repair, pore-forming toxins, infectious diseases, muscular dystrophy, degenerative diseases

## Abstract

The plasma membrane of mammalian cells is susceptible to disruption by mechanical and biochemical damages that frequently occur within tissues. Therefore, efficient and rapid repair of the plasma membrane is essential for maintaining cellular homeostasis and survival. Excessive damage of the plasma membrane and defects in its repair are associated with pathological conditions such as infections, muscular dystrophy, heart failure, diabetes, and lung and neurodegenerative diseases. The molecular events that remodel the plasma membrane during its repair remain poorly understood. In the present work, we report the development of a quantitative high-throughput assay that monitors the efficiency of the plasma membrane repair in real time using a sensitive microplate reader. In this assay, the plasma membrane of living cells is perforated by the bacterial pore-forming toxin listeriolysin O and the integrity and recovery of the membrane are monitored at 37°C by measuring the fluorescence intensity of the membrane impermeant dye propidium iodide. We demonstrate that listeriolysin O causes dose-dependent plasma membrane wounding and activation of the cell repair machinery. This assay was successfully applied to cell types from different origins including epithelial and muscle cells. In conclusion, this high-throughput assay provides a novel opportunity for the discovery of membrane repair effectors and the development of new therapeutic compounds that could target membrane repair in various pathological processes, from degenerative to infectious diseases.

## Introduction and limitations of current models

The repair of the plasma membrane is a fundamental process that maintains cell homeostasis, prevents the loss of difficult to replace cells (e.g., cardiac myocytes or neurons) and eliminates the need for replacing frequently injured cells. Mechanical stress and molecules that can directly damage the plasma membrane are major causes of cell injuries. Membrane injuries due to mechanical wounding frequently occur in contractile tissues (McNeil and Khakee, 1992; Demonbreun and McNally, 2016; Cong et al., 2017). Pore-forming proteins released by pathogens and immune system effectors also severely compromise the integrity of the plasma membrane during infection and inflammation (Morgan et al., 1986; Geeraerts et al., 1991; Gonzalez et al., 2008). In response to these various sources of injuries, eukaryotic cells rapidly repair their plasma membrane (McNeil and Steinhardt, 1997, 2003; Blazek et al., 2015; Demonbreun and McNally, 2016). Excessive plasma membrane damage and defects in its repair are associated with diverse pathological conditions such as muscular dystrophy, heart failure, diabetes, lung and neurodegenerative diseases (Clarke et al., 1995; Bansal et al., 2003; Han et al., 2007; Idone et al., 2008; Cai et al., 2009; Howard et al., 2011a; Blazek et al., 2015; Fernandez-Perez et al., 2016; Peters et al., 2016; Cong et al., 2017).

The objective of this work was to develop a high-throughput, microplate-based assay to assess the repair efficiency of the plasma membrane of mammalian cells. Different models for the plasma membrane repair process have been proposed; they all consider that the influx of extracellular Ca^2+^, through the site of injury, is a trigger for activation of the repair process. The proposed repair processes include the decrease in membrane surface tension via dissociation of the cortical F-actin network, patching of the membrane lesions by fusion of intracellular vesicles with the plasma membrane, regrowth and contraction of F-actin at the edges of the lesion to close the wound, removal of membrane lesions by endocytosis, shedding of the lesion-containing membrane, and exocytosis of enzymes that degrade the agents responsible for membrane attack (e.g., pore-forming toxins) (Bi et al., 1995; McNeil and Steinhardt, 1997; Terasaki et al., 1997; Togo et al., 2000; Arnett et al., 2014; Moe et al., 2015). Despite experimental evidence that supports these repair processes there are still important questions that remain unanswered. It is poorly understood if several or all of the proposed processes function together to ensure an extremely rapid and efficient recovery, if some of those processes operate in a cell-type-dependent fashion, or if the use of one or several of these processes depends upon the nature and extent of the plasma membrane wound. Furthermore, only a few Ca^2+^ sensors such as synaptotagmin, the annexins and more cell type-specific proteins such as dysferlin and TRIM72/MG53 have been identified as key players in the repair processes, which leaves many additional effector molecules left to be identified (Chakrabarti et al., 2003; Weisleder et al., 2009; Draeger et al., 2011; Defour et al., 2014a). Therefore, it is necessary to develop additional experimental tools to identify the membrane repair effectors and refine our understanding of the repair processes. Such tools could also be useful for the development of new therapeutic compounds that target membrane repair defects in various disease states.

Several approaches are used to model the mechanical cell wounding that normally occurs under physiologically strenuous conditions in skeletal muscles, heart, lungs, or intestines. These approaches consist of inducing contraction or stretching of a cell monolayer grown on a flexible surface, cell scraping from the dish, or creating membrane abrasions with glass beads (Liu et al., 1995; Belete et al., 2010; Mellgren, 2010; Howard et al., 2011b; Defour et al., 2014b). The membrane disruptions can be relatively large (>100 nm in diameter) and lead to a massive influx of extracellular Ca^2+^. However, controlling the size of the membrane lesions by these approaches is challenging and cells in the population may not be uniformly damaged. Alternatively, micro-needle insertion and laser-induced perforation make a site-specific wound of desired size that can be more precisely controlled and therefore, the reproducibility of the wound is high (Steinhardt et al., 1994; Terasaki et al., 1997; Jimenez et al., 2015). However, these methods work on a single cell and are not amenable to high-throughput screening.

Plasma membrane wounding can also be achieved by adding pore-forming agents such as bacterial toxins to the cell culture medium. The size of the membrane pores can vary from 1 to 50 nm depending upon the toxin. Small pores such as those formed by aerolysin (produced by *Aeromonas* species) do not form efficient Ca^2+^ channels and are not well suited for the study of plasma membrane repair that requires the influx of extracellular Ca^2+^. In contrast, a massive influx of extracellular Ca^2+^ occurs in cells perforated by the very large (30 to 50 nm) pores of the cholesterol-dependent cytolysins (CDCs) 191 family (Repp et al., 2002; Dunstone and Tweten, 2012; Cajnko et al., 2014; Tweten et al., 2015). CDCs are produced by numerous bacterial species and constitute powerful tools for studying membrane resealing. Membrane wounding with CDCs can be effectively used to study cell repair at the cell population level with high reproducibility (Corrotte et al., 2015). Most CDCs use cholesterol as a receptor and therefore can perforate the plasma membrane of any mammalian cells. The CDC streptolysin O produced by *Streptococcus pyogenes* was successfully used to gain insight into the membrane repair processes (Idone et al., 2008). In the present work, we used listeriolysin O (LLO), the CDC secreted by the foodborne pathogen *Listeria monocytogenes* as a tool to perforate mammalian cells (Seveau, 2014).

To establish the efficiency of plasma membrane repair, most approaches rely on the quantification of plasma membrane integrity using membrane impermeant dyes. Those include Trypan blue, propidium iodide, and FM-dyes, which can penetrate wounded cells leading to a change in cell color or fluorescence (Cochilla et al., 1999; Defour et al., 2014b). Trypan blue has been routinely used for distinguishing live from dead cells, but it lacks the sensitivity required for membrane repair assays (Tran et al., 2011). Propidium iodide (PI) generates quantifiable fluorescence upon binding to nucleic acids inside cells. Membrane selective lipophilic FM dyes (FM4-64 and FM1-43), which fluorescence quantum yields increase in the hydrophobic environment of the phospholipid bilayer, only label the plasma membrane of intact cells, but generate high fluorescence when they enter damaged cells and bind the membranes of all intracellular organelles. While both FM dyes and PI can be utilized for live-cell imaging, PI does not label intact cells (as FM dyes do) providing a more accurate measurement of cell integrity. In the present work, we used PI to quantify the efficiency of membrane repair.

Quantitative fluorescence microscopy and flow-cytometry can be used to measure the uptake of fluorescent dyes by damaged cells. The advantage of flow cytometry is the rapid measurement of large cell populations (Idone et al., 2008) and it is well adapted for suspended cells. However, many studies on membrane repair involve adherent mammalian cells, which require the detachment of cells prior to the experiment, thus compromising the properties of the plasma membrane that can seriously impact the experimental measurements. Also, trypsin treatment likely alters the repair capacity of cells as it digests many surface proteins. Quantitative fluorescence microscopy analysis of fixed and living cells has been a useful approach for studying the repair mechanisms (Defour et al., 2014b). In live-cell imaging, spatiotemporal dynamics of molecular events can be directly monitored in cells expressing fluorescent proteins or labeled with fluorescent dyes. However, microscopy-based approaches are less amenable to high-throughput analyses. Therefore, the present assay uses a temperature-controlled plate reader to quantify PI fluorescence intensities in living cells cultured in 96-well plates, allowing for high-throughput temporal analyses at the cell population level.

## Materials and methods

### Reagents and recombinant listeriolysin O

For wounding the plasma membrane, cells were exposed to recombinant six His-tagged-listeriolysin O (LLO), purified as previously described (Vadia et al., 2011). Hanks balanced salts (HBSS) without Ca^2+^ and Mg^2+^, propidium iodide (PI), EGTA, and cytochalasin D were acquired from Sigma Aldrich. Assay buffer M1 consisted of HBSS supplemented with 0.5 mM MgCl_2_, 1.2 mM CaCl_2_, 10 mM HEPES, 25 mM Glucose, pH 7.4. Assay buffer M2 consisted of HBSS supplemented with 0.5 mM MgCl_2_, 10 mM HEPES, and 25 mM Glucose, pH 7.4. When indicated, cells were washed in M2 supplemented with 5 mM EGTA.

### Mammalian cell cultures

We selected two mammalian cell lines, HeLa (ATCC #CCL-2) and C2C12 (ATCC #CRL-1772), which have been frequently used in studies assessing the mechanisms of membrane repair. HeLa cells are of human epithelial origin and were used to study membrane repair following damage by either mechanical or biological injuries (Idone et al., 2008; Howard et al., 2011b). C2C12 cells are immortalized murine myocytes used in many studies of muscle cell biology (Howard et al., 2011b) and membrane repair (Demonbreun and McNally, 2016). The human cervical epithelial HeLa cell line was grown in minimum essential medium (MEM) (+) Earle's salts and L-glutamine (Invitrogen), supplemented with 10% heat inactivated fetal bovine serum (HI-FBS; Atlanta Biologicals), 0.1 mM non-essential amino acids, 1 mM sodium pyruvate, 100 U/ml penicillin, and 100 μg/ml streptomycin (Invitrogen). The mouse muscle myoblast C2C12 cell line was grown in Dulbecco's Modified Eagle's Medium (DMEM) with high glucose (Thermo Fisher Scientific) supplemented with 10% HI-FBS and 100 units/ml penicillin and 100 μg/ml streptomycin. Mammalian cells were maintained at 37°C in 5% CO_2_ atmosphere. Since the extent of wounding/repair depends on the number of cells and dosage of the toxin, a tight control over these two parameters is necessary to obtain reproducible results. Therefore, for a successful assay, it should be ensured that cells are plated at similar density (70–80% confluence) in all wells and experiments.

### Plate reader and 96-well plates

The assays involved cells cultured in 96-well plates and kinetics were performed at 37°C for 30 min with fluorescence measurements at 5 min time intervals. Both the duration and time intervals can be modified based on experimental needs. The minimum time interval for measurement of a full 96-well-plate with the Spectra Max i3x Multi-Mode Detection Platform (Molecular devices) is 30 s. Two different plate types were used in these studies. Plate 1: Corning® 96-well flat clear bottom black polystyrene TC-treated microplates, individually wrapped, with lid and sterile (#3603). Plate 2: Nunc™ 96-well polystyrene round bottom sterile plates (#262162).

### Kinetic assay to monitor membrane injury and repair

Cells were plated in a 96-well plate (plate 1) in triplicate for each experimental condition, in 200 μl of their respective culture medium (HeLa: 2.5 × 10^4^ cells/well or C2C12: 1 × 10^4^ cells/well). The following day, cells in plate 1 were pre-incubated with the indicated concentrations of cytochalasin D (Sigma) or equivalent amounts of the DMSO vehicle for 10 min at 37°C. Cytochalasin D, or control DMSO, was maintained at the same concentration in the assay buffers (**Figure 4** only). To perform the kinetic assay (all figures), cells were washed with pre-warmed medium (37°C) using a multichannel pipette as follows: two washes with 200 μl M1, followed by the addition of 100 μl M1 (for experimental conditions carried out in the presence of Ca^2+^); or one wash with 200 μl M2 supplemented with 5 mM EGTA, one wash with 200 μl M2, followed by the addition of 100 μl M2 (for experimental conditions carried out without extracellular Ca^2+^). The assay reagents were pre-loaded at 4°C in a separate 96-well plate (plate 2, with same experimental setting as plate 1) as follows: 100 μl M1 (or M2) containing 120 μM PI (4X) plus 100 μl M1 (or M2) containing, or not, LLO (4X). Using a multichannel pipette, 100 μl were transferred from plate 2 to plate 1, gently homogenized, and plate 1 was immediately placed in the plate reader at 37°C. Fluorescence was measured as the average of 6 upward readings at 5 min time intervals for 30 min. The excitation and emission wavelengths were 535 and 617 nm, respectively, with a 15 nm bandwidth. Immediately after the kinetic assay, phase-contrast and fluorescence images were acquired with a 4X air objective within the plate reader. For data analysis, cells incubated with PI in M1 or M2 (in the presence of corresponding drugs or vehicle when appropriate), but in the absence of LLO, served as controls to establish the fluorescence intensity baselines for each experimental condition and at each time point of the kinetics. The respective baselines were subtracted from the raw data obtained with cells incubated with LLO. Presented data were the average of three or more independent experiments; each performed in triplicate on different days, and were expressed as the mean ± Standard Error of the Mean (SEM).

### Statistical analyses

The baseline normalized individual data was first log 10 transformed to reduce skewness and variance, and then the mean of the triplicates of each independent experiment was used for analysis with linear mixed effects models to take account of the correlation among the observations from the same independent replicate. In order to test whether the speed of fluorescence intensity change over time is significantly different between the lowest concentration (0.1 nm) and other higher concentrations within the same treatment condition, as well as the same concentration between the two conditions (M1 and M2), trend of fluorescence intensity change over time was compared from the linear mixed models. In addition, the mean fluorescence intensity averaged across the measurement time was also compared between the aforementioned groups to investigate whether the higher concentrations induced overall higher fluorescence intensity than the 0.1 nM concentration and whether there is difference between M1 and M2 conditions for the same concentration used. The fluorescence intensity was also compared among the aforementioned groups at time = 30 min using an ANOVA model. Holm's procedure was used to adjust for multiple testing and the adjusted *p* < 0.05 was considered as significant.

## Results

### High-throughput analysis of membrane wounding and repair: sensitivity and reproducibility of the assay

**Figure 1:**
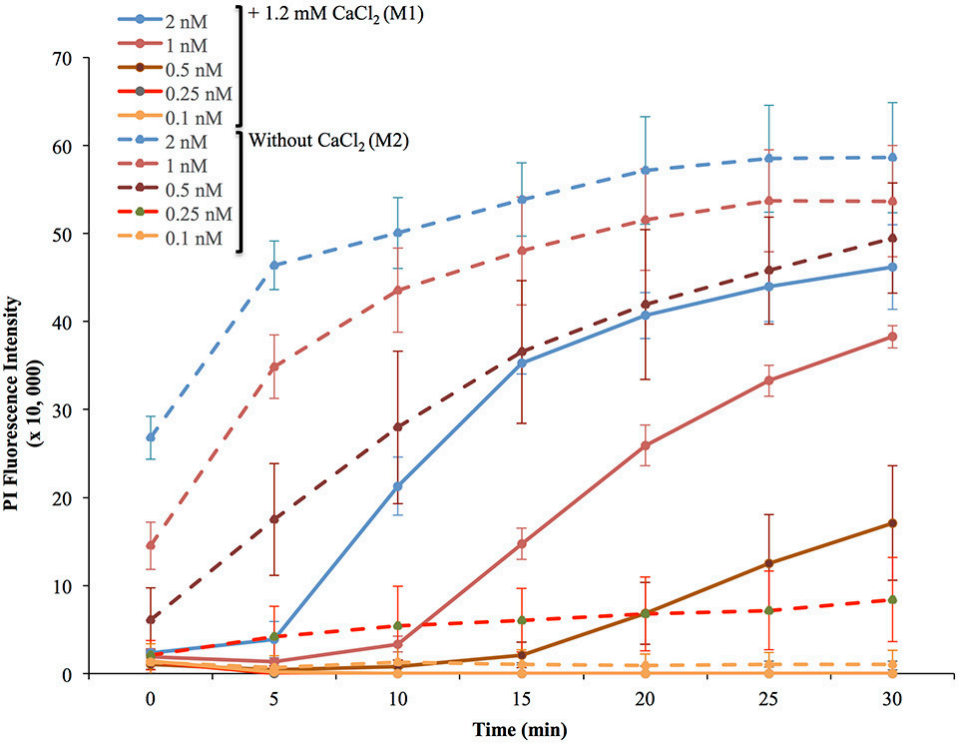
Representative experiment of membrane wounding by LLO and repair kinetics in HeLa cells. HeLa cells were exposed to the indicated concentrations of LLO in M1 (+ 1.2 mM CaCl_2_, solid lines) or M2 (without CaCl_2_, dashed lines) containing 30 μM PI and incubated in the plate reader at 37°C for 30 min. Fluorescence intensities were measured every 5 min. The baseline fluorescence levels of control cells incubated without LLO, at each time point in M1 and in M2, were subtracted from the values obtained with cells incubated with LLO. Data are the average fluorescence intensities expressed in arbitrary unit ± standard deviations (S.D.) of triplicates for each experimental condition.

**Figure 2:**
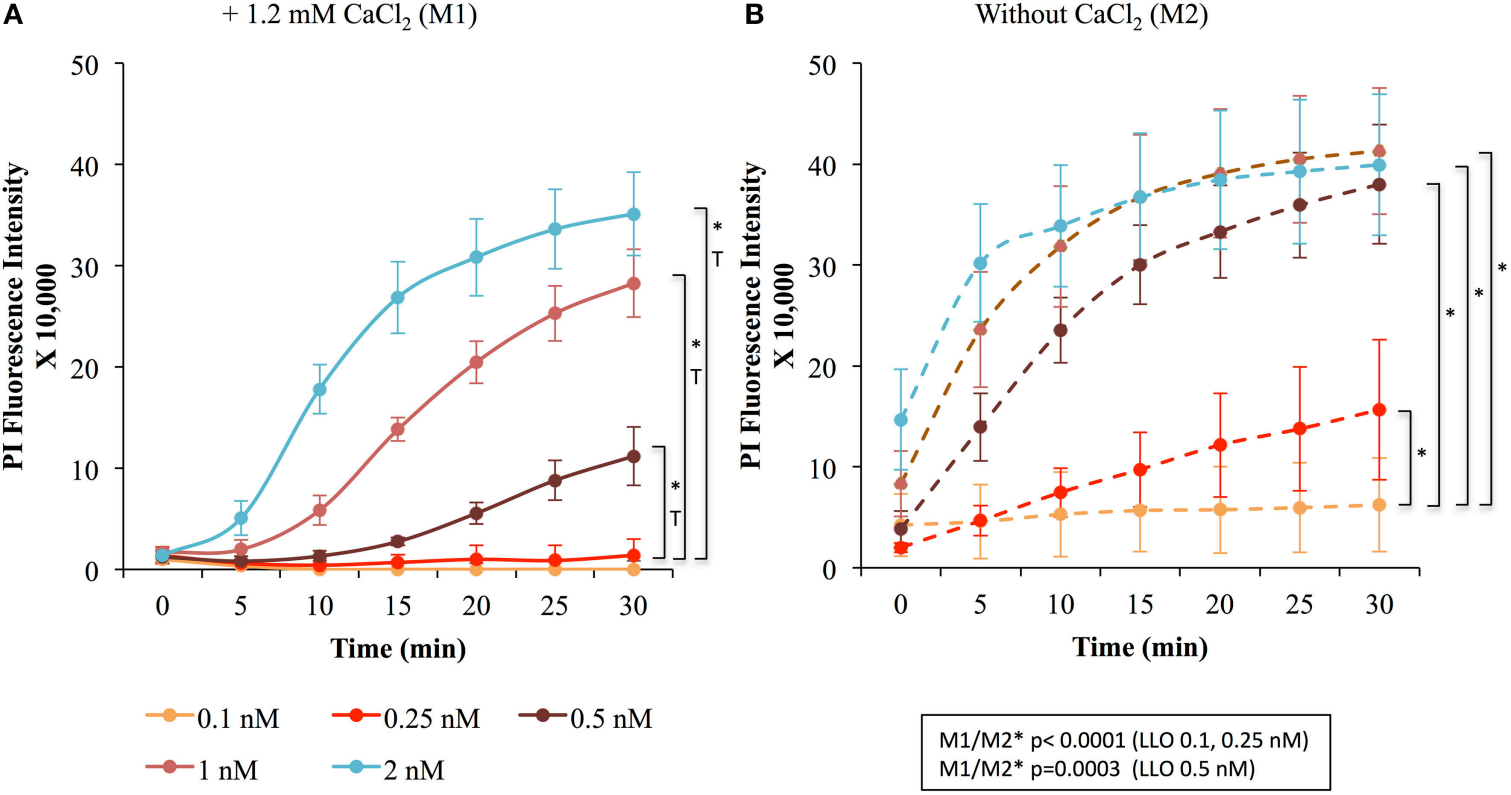
LLO induces dose-dependent membrane wounding and repair in HeLa cells. HeLa cells were exposed to the indicated concentrations of LLO in M1 (solid lines) **(A)** or M2 (dashed lines) **(B)** supplemented with 30 μM PI and incubated in the plate reader for 30 min at 37°C. At each time point in M1 **(A)** and M2 **(B)**, the baseline fluorescence level of control cells incubated without LLO were subtracted from the values obtained with cells incubated with LLO. Data are the average fluorescence intensities of four independent experiments, with the error bars representing the standard error of the mean (SEM). Statistical analyses correspond to the trend comparison (T, *p* < 0.0005) and the mean fluorescence intensities averaged across all time points (^*^, *p* < 0.0005). Those analyses compared the different concentrations of LLO in M1 **(A)** or in M2 **(B)** and compared data obtained in M1 vs. M2 (M1/M2) at a given concentration of LLO (showed in the squared box). Statistical analyses are also recapitulated in Tables 1A,B.

**Figure 3:**
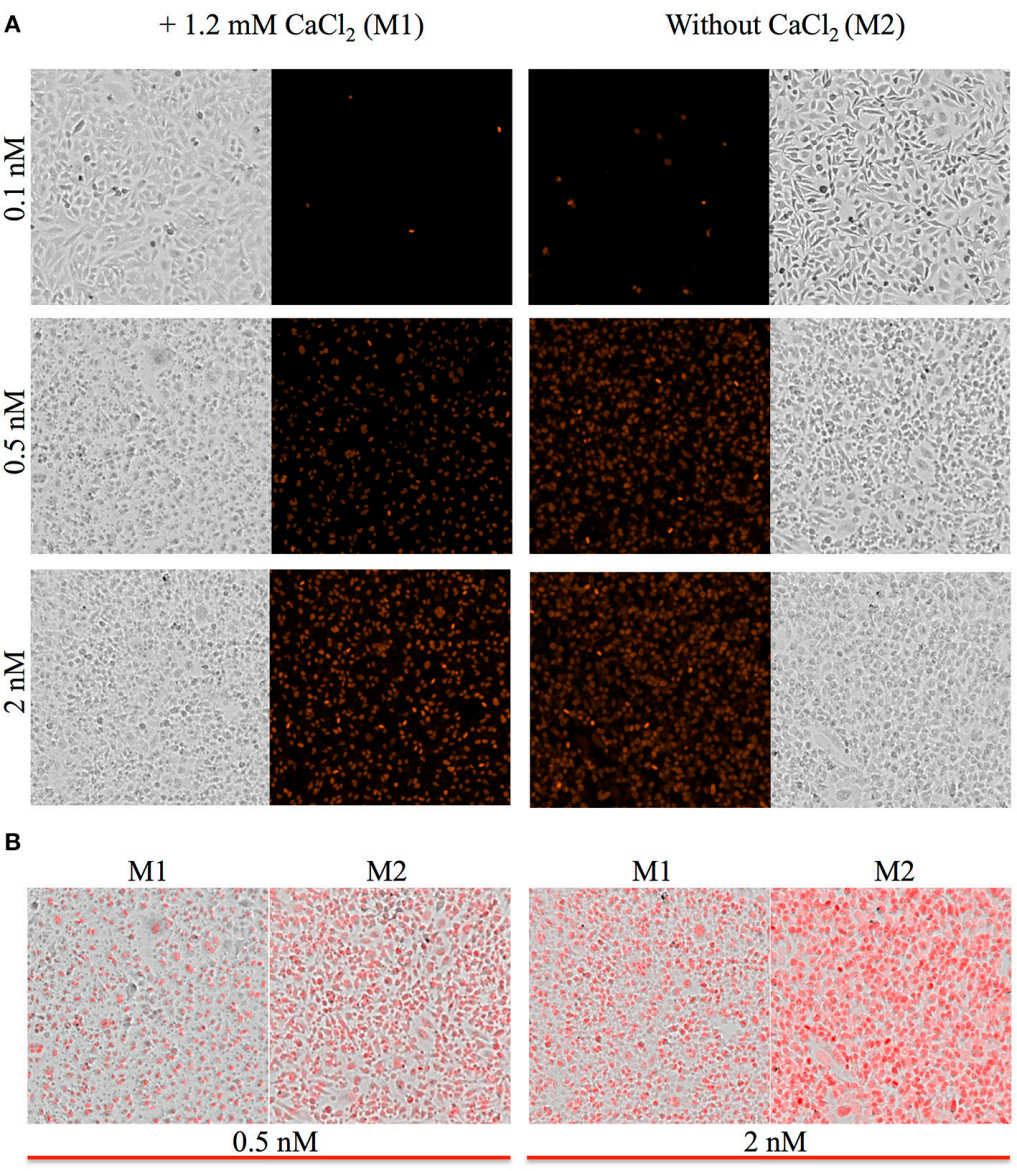
Representative images of HeLa cells. Phase contrast and fluorescence images of HeLa cells were acquired with a 4X objective located within the plate reader at the end of the kinetic assay corresponding to time the point 30 min. **(A)** Cells were incubated in M1 or M2 with the indicated concentrations of LLO. All fluorescence intensities are presented using the same intensity scale. **(B)** Overlay of phase contrast and fluorescence images are presented to facilitate the comparison between the M1 and M2 experimental conditions.

To measure the efficiencies of plasma membrane wounding and repair following exposure to LLO (0.1–2 nM), we incubated HeLa cells with the fluorescent dye propidium iodide (PI) in media supplemented (M1), or not (M2), with CaCl_2_. In the absence of extracellular Ca^2+^ (M2), cells cannot repair their plasma membrane and consequently, the PI fluorescence intensities reflected the extent of cell wounding by LLO. In the presence of extracellular Ca^2+^ (M1), cells can undergo repair and the PI fluorescence intensities reflected the contributions of both cell wounding by LLO and repair. Importantly, formation of the LLO pore is a Ca^2+^-independent process (Arnett et al., 2014). Each experimental condition was carried out in triplicate and a representative experiment is shown in Figure 1. In M1, at each LLO concentration, the rise in PI fluorescence over time reflected the accumulation of PI that could enter through the toxin pores. As expected, for each LLO concentration, higher fluorescence intensities were recorded in M2 compared to M1, which is evidence of plasma membrane repair in M1. The rise in PI fluorescence with increasing concentrations of LLO reflected the dose-dependent activity of LLO. The relatively small standard deviations (S.D.) within triplicates for each experimental condition reflected the intra-assay reproducibility. Reproducibility of the assay was further supported by the fact that when multiple independent experiments were averaged (*n* = 4), the values of the standard error of the mean (S.E.M.) were of moderate amplitudes (Figure 2). We also measured the standard deviations corresponding to data presented in Figure 2 (Supplemental Figure 1). Statistical analyses established that there is a LLO dose response effect in both M1 and M2 conditions: with the increase in LLO concentration, there is increased fluorescence intensity averaged across time and conditions (*p* = 0.0002). The increase in fluorescence intensity over time was much more pronounced in M1, with statistical significance between each successive concentrations of LLO from 0.1 to 1 nM except dose 0.25 (see Table 1A) based on the trend (slope) analysis. In M2, the increase in fluorescence intensity over time was not statistically significant between LLO 0.1 nM and any of the higher concentrations (*p* > 0.05, Table 1A). This likely is due to rapid and excessive damages in the absence of repair of the plasma membrane. However, the fluorescence intensities averaged across time were significantly higher comparing the higher doses to 0.1 nM for both M1 and M2 conditions (*p* < 0.05, Table 1B). We also compared data obtained between M1 and M2 for each concentration of LLO. A statistically significant difference between M1 and M2 was observed at the concentrations of 0.1, 0.25, and 0.5 nM when considering the fluorescence intensities averaged across all time points after Holm's procedure adjustment of multiple comparisons (Figure 2 and Table 1B). However, at LLO 1 and 2 nM differences between cells incubated in M1 vs. M2 were not significant, likely due to the extensive damage inflicted by the toxin, leading to ineffective repair at those concentrations. From these experiments, we conclude that in HeLa cells, the concentrations of LLO that are best suited for the study of membrane repair range from 0.1 to 0.5 nM. Figure 3 includes representative phase-contrast and fluorescence images of HeLa cells exposed to increasing concentrations of LLO in M1 and M2. We can appreciate that the cell density remained unaffected in all experimental conditions, which is crucial for the validity of the assay. Additionally, the images allow one to appreciate differences between M1 and M2: (i) difference in extent of cell damage and (ii) difference in the number of damaged cells, which are both higher in M2. Together, these results indicate that this assay can effectively produce varying degrees of membrane damage in mammalian cells while allowing simultaneous monitoring of the repair efficiencies of the cell population.

**Table 1A:** Fluorescence intensity change over time (trend) was compared between LLO concentrations higher than 0.1 nM and that of 0.1 nM for M1 and M2 from a linear mixed effects model.

**Trend analysis of fluorescence increase over time for HeLa cells**				
	**Trend estimate**	***p*****-values**	**95% CI of trend**	
Dose Effect	2.7087	0.0002	1.2845	4.1328
M1 comparing LLO 2 to 0.1	0.1487	<0.0001	0.09871	0.1986
M1 comparing LLO 1 to 0.1	0.1448	<0.0001	0.09480	0.1947
M1 comparing LLO 0.5 to 0.1	0.1557	<0.0001	0.1058	0.2057
M1 comparing LLO 0.25 to 0.1	0.03857	0.1297	−0.01140	0.08853
M2 comparing LLO 2 to 0.1	0.006204	0.8070	−0.04376	0.05617
M2 comparing LLO 1 to 0.1	0.01519	0.5498	−0.03477	0.06516
M2 comparing LLO 0.5 to 0.1	0.02558	0.3143	−0.02438	0.07554
M2 comparing LLO 0.25 to 0.1	0.01501	0.5546	−0.03495	0.06497

*Holm's procedure was used to adjust for multiple comparisons*.

**Table 1B:** Mean fluorescence intensities averaged across time were compared between LLO concentrations higher than 0.1 and 0.1 nM within M1 and M2, as well as between M1 and M2 for the same dose, where the estimate is the difference between condition 1 LLO dose 1 and condition 2 LLO dose 2.

**Comparison of fluorescence intensity averaged across time for HeLa cells**							
**Condition 1**	**LLO 1 (nM)**	**Condition 2**	**LLO 2 (nM)**	**Estimate**	***p*****-values**	**95% CI of estimate**	
M1	0.1	M1	0.25	−0.6610	0.0097	−1.1606	−0.1614
M1	0.1	M1	0.5	−2.4562	<0.0001	−2.9559	−1.9566
M1	0.1	M1	1	3.0816	<0.0001	2.5820	3.5813
M1	0.1	M1	2	3.2959	<0.0001	2.7963	3.7955
M2	0.1	M2	0.25	−0.6223	0.0148	−1.1220	−0.1227
M2	0.1	M2	0.5	−0.7325	0.0042	−1.2322	−0.2329
M2	0.1	M2	1	1.0507	<0.0001	0.5511	1.5503
M2	0.1	M2	2	1.1092	<0.0001	0.6096	1.6089
M1	0.1	M2	0.1	−2.6650	<0.0001	−3.1646	−2.1654
M1	0.25	M2	0.25	−2.6263	<0.0001	−3.1260	−2.1267
M1	0.5	M2	0.5	−0.9413	0.0003	−1.4409	−0.4417
M1	1	M2	1	−0.6341	0.0131	−1.1337	−0.1344
M1	2	M2	2	−0.4784	0.0605	−0.9780	0.02126

### Validation of the assay to the identification of effectors of the repair machinery

**Figure 4:**
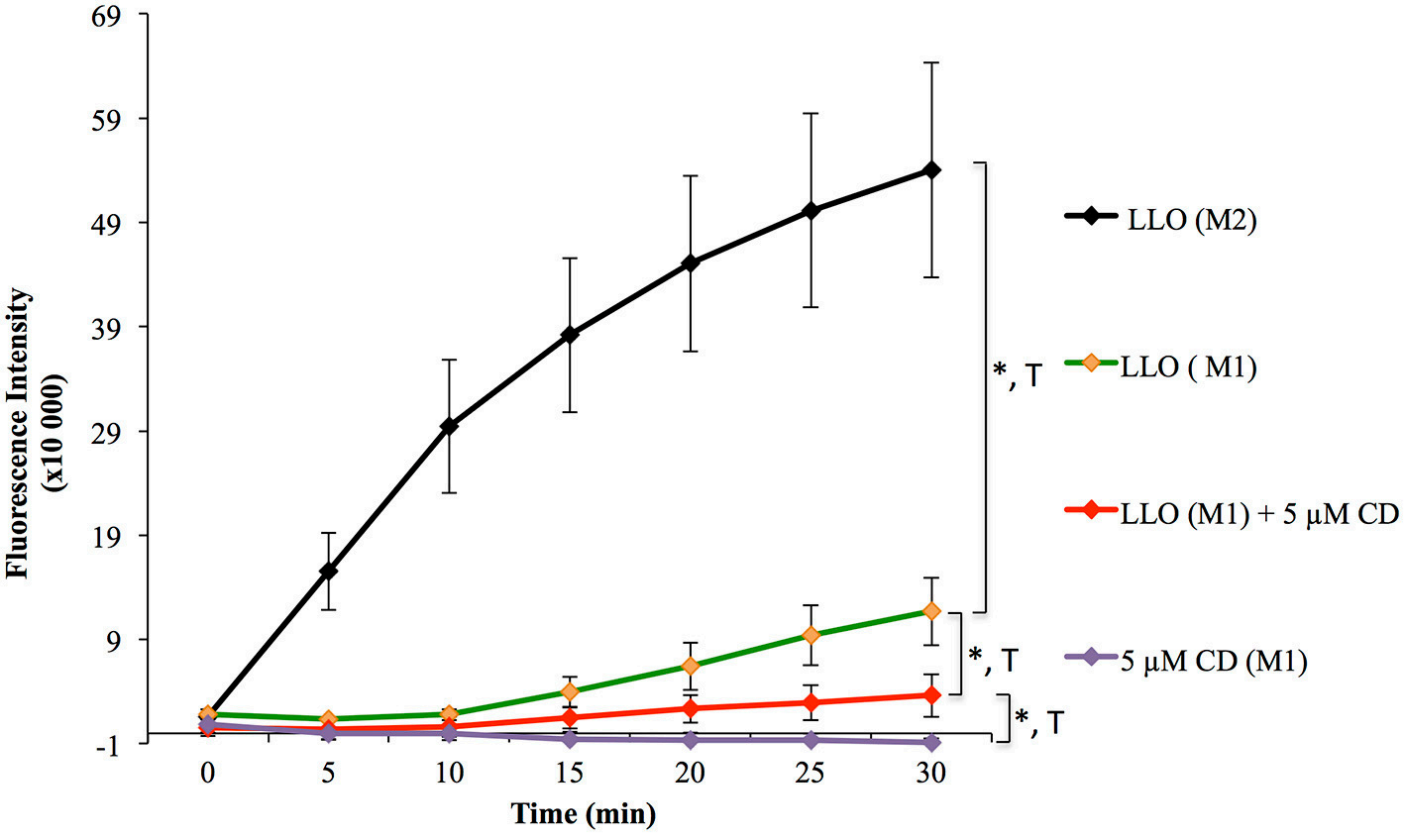
Effect of F-actin disassembly on plasma membrane repair. HeLa cells were pre-incubated in the presence of 5 μM cytochalasin D (CD, stock solution was stored in DMSO) for 10 min at 37°C, and the drug was maintained at the same concentration throughout the assay. Cells were exposed to 0.5 nM LLO, or not, in M1 or M2 supplemented with 30 μM PI. Control cells were incubated with a dilution of vehicle (DMSO) similar to cells treated with CD. Data are the average of four independent experiments, each performed in triplicate, and the error bars represent the standard error of mean (SEM). Statistical analyses correspond to the trend comparison (T, *p* < 0.0005) and the mean fluorescence intensities averaged across all time points (^*^, *p* < 0.0005). Statistical analyses are also recapitulated in Tables 2A,B.

To validate the use of this assay for the identification of molecules that affect the repair machinery, we treated cells with the drug cytochalasin D (CD). This drug induces the disassembly of F-actin by binding to the barbed end of actin filaments. Cell treatment with low concentrations of CD has been shown to slightly enhance the membrane repair efficiency of cells exposed to LLO (Vadia et al., 2011). Therefore, we thought that if our assay could detect a subtle change in the repair efficiency upon cell treatment with CD, this would validate the sensitivity of the assay. HeLa cells, pre-treated or not with CD, were exposed to 0.5 nM LLO and were monitored in the plate reader at 37°C for 30 min (Figure 4). Data showed that F-actin disassembly facilitates the repair process, as expected. Importantly, there was a statistically significant difference between cells exposed to LLO in the presence vs. the absence of CD and between cells exposed to LLO in the presence or absence of Ca^2+^ (Figure 4 and Table 2). In conclusion, this assay can be used to quantitatively evaluate the role of effectors that are involved in repair either by promoting (presence of extracellular Ca^2+^) or preventing (subcortical F-actin cytoskeleton) cell resealing.

**Table 2A:** Fluorescence intensity change over time (trend) was compared between the indicated experimental conditions from a linear mixed effects model.

**Trend analysis of fluorescence increase over time for HeLa cells**					
**Experimental conditions**	**Estimate**	**Standard error**	***p*****-values**	**95% CI of trend**	
Comparing LLO (M2) to LLO (M1)	−0.00710	0.02206	0.7478	−0.0507	0.03647
Comparing LLO (M1) and LLO (M1) + CD	0.1088	0.02206	<0.0001	0.06521	0.1524
Comparing LLO (M1) + CD and (M1) + CD	0.09729	0.02206	<0.0001	0.05372	0.1409

*Holm's procedure was used to adjust for multiple comparisons*.

**Table 2B:** Mean fluorescence intensities averaged across time were compared between the experimental conditions 1 and 2, where the estimate is the difference between condition 1 and condition 2.

**Comparison of fluorescence intensity averaged across time for Hela cells**					
**Condition 1**	**Condition 2**	**Estimate**	***p*****-values**	**95% CI of estimate**	
LLO (M1)	LLO (M2)	−0.7887	0.0005	−1.2244	−0.3530
LLO (M1)	LLO (M1) + CD	1.1448	<0.0001	0.7091	1.5805
LLO (M1) + CD	(M1) + CD	1.4408	<0.0001	1.0051	1.8765

### Validation of the assay for the study of muscle cell repair

**Figure 5:**
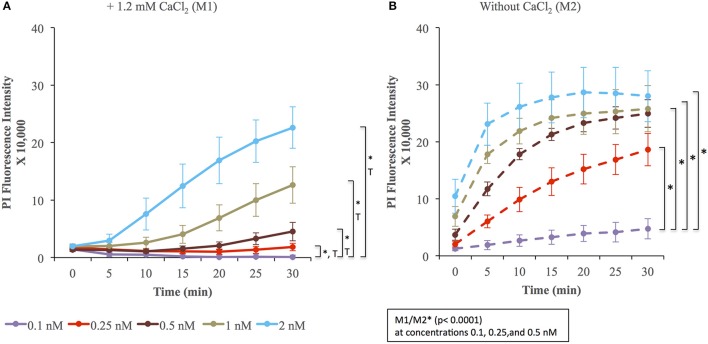
Membrane wounding and repair in muscle cells. C2C12 cells were exposed to the indicated concentrations of LLO in M1 **(A)** and M2 **(B)** supplemented with 30 μM PI for 30 min at 37°C in the plate reader. Fluorescence intensities were measured every 5 min. The baseline fluorescence level, at each time point in M1 **(A)** and M2 **(B)**, of control cells incubated without LLO were subtracted from the values obtained with cells incubated with LLO. Data are the average fluorescence intensities expressed in arbitrary unit ± standard error of the mean (S.E.M.) of four independent experiments. Statistical analyses correspond to the trend comparison (T, *p* < 0.0001, except for concentration 0.25 nM *p* = 0.02) and the mean fluorescence intensities averaged across all time points (^*^, *p* < 0.0001). Those analyses compared the different concentrations of LLO in M1 **(A)** or in M2 **(B)** and compared data obtained in M1 vs. M2 (M1/M2) at a given concentration of LLO (showed in the squared box). Statistical analyses are also recapitulated in Tables 3A–C.

We evaluated the applicability of the assay to membrane repair in muscle cells. We exposed C2C12 cells to various concentrations of LLO, as performed previously with Hela cells. Data presented in Figure 5 (and Supplemental Figure 1) showed that C2C12 cells display both similar sensitivity to wounding by LLO and similar extent of membrane resealing when compared with HeLa cells (Figure 2). Statistical analyses established that there is a LLO dose response effect in both M1 and M2 conditions as in HeLa cells: with the increase in LLO concentration, there is increased fluorescence intensity averaged across time and conditions (*p* < 0.0001; Table 3A). Importantly, significantly higher mean fluorescence intensity were measured in M2 when compared to M1 at LLO concentrations 0.1, 0.25, and 0.5 nM, but not at 1 and 2 nM after Holm's procedure adjustment of multiple comparisons (Tables 3A,B). This reflects that cells efficiently repaired their plasma membrane at the lower concentrations of LLO, but not at the higher concentrations 1 and 2 nM. This assay can also be used as an end point assay by measuring the fluorescence intensities at 30 min (Table 3C).

**Table 3A:** Fluorescence intensity change over time (trend) was compared between LLO concentrations higher than 0.1 nM and that of 0.1 nM for M1 and M2 from a linear mixed effects model.

**Trend analysis of fluorescence increase over time for C2C12 cells**				
	**Trend estimate**	***p*****-values**	**95% CI of trend estimate**	
Dose effect	2.4171	<0.0001	1.3080	3.5262
M1 comparing LLO 2 to 0.1	0.1072	<0.0001	0.06825	0.1461
M1 comparing LLO 1 to 0.1	0.09803	<0.0001	0.05912	0.1369
M1 comparing LLO 0.5 to 0.1	0.09391	<0.0001	0.05501	0.1328
M1 comparing LLO 0.25 to 0.1	0.04633	0.0198	0.007420	0.08524
M2 comparing LLO 2 to 0.1	−0.01475	0.4559	−0.05366	0.02416
M2 comparing LLO 1 to 0.1	−0.01224	0.5360	−0.05115	0.02667
M2 comparing LLO 0.5 to 0.1	−0.00343	0.8624	−0.04234	0.03548
M2 comparing LLO 0.25 to 0.1	0.001384	0.9442	−0.03753	0.04029

*Holm's procedure was used to adjust for multiple comparisons*.

**Table 3B:** Mean fluorescence intensities averaged across time were compared between LLO concentrations higher than 0.1 and 0.1 nM within M1 and M2, as well as between M1 and M2 for the same dose, where the estimate is the difference between condition 1 LLO dose 1 and condition 2 LLO dose 2.

**Comparison of fluorescence intensity averaged across time for C2C12 cells**							
**Condition 1**	**LLO 1 (nM)**	**Condition 2**	**LLO 2 (nM)**	**Estimate**	***p*****-values**	**95% CI of estimate**	
M1	0.1	M1	0.25	−1.2962	<0.0001	−1.6853	−0.9071
M1	0.1	M1	0.5	−1.4885	<0.0001	−1.8776	−1.0994
M1	0.1	M1	1	−2.0664	<0.0001	−2.4555	−1.6773
M1	0.1	M1	2	−2.3735	<0.0001	−2.7626	−1.9845
M2	0.1	M2	0.25	−0.7891	<0.0001	−1.1782	−0.4000
M2	0.1	M2	0.5	−1.0147	<0.0001	−1.4038	−0.6256
M2	0.1	M2	1	−1.1049	<0.0001	−1.4940	−0.7158
M2	0.1	M2	2	−1.1723	<0.0001	−1.5614	−0.7832
M1	0.1	M2	0.1	−1.6531	<0.0001	−2.0422	−1.2640
M1	0.25	M2	0.25	−1.1460	<0.0001	−1.5351	−0.7569
M1	0.5	M2	0.5	−1.1793	<0.0001	−1.5684	−0.7902
M1	1	M2	1	0.6915	0.0005	−1.0806	−0.3024
M1	2	M2	2	−0.4518	0.1230	−0.8409	−0.06269

**Table 3C:** Mean fluorescence intensities averaged across time were compared between various LLO concentrations between M1 and M2, where the estimate is the difference between condition 1 LLO dose 1 and condition 2 LLO dose 2.

**Comparison of fluorescence intensities at 30 min for C2C12 cells**							
**LLO 1 (nM)**	**Condition 1**	**LLO 2 (nM)**	**Condition 2**	**Estimate**	***p*****-values**	**95% CI of estimate**	
0.1	M1	0.25	M1	−2.2701	0.0001	−3.3177	−1.2225
0.1	M1	0.5	M1	−2.6468	<0.0001	−3.6944	−1.5991
0.1	M1	1	M1	−3.1918	<0.0001	−4.2394	−2.1441
0.1	M1	2	M1	−3.4781	<0.0001	−4.5257	−2.4305
0.1	M2	0.25	M2	−0.7081	0.1769	−1.7557	0.3396
0.1	M2	0.5	M2	−0.8450	0.1095	−1.8926	0.2027
0.1	M2	1	M2	−0.8510	0.1071	−1.8986	0.1966
0.1	M2	2	M2	−0.8843	0.0947	−1.9320	0.1633
0.1	M1	0.1	M2	−2.6866	<0.0001	−3.7342	−1.6389
0.25	M1	0.25	M2	−1.1245	0.0364	−2.1722	−0.07690
0.5	M1	0.5	M2	−0.8848	0.0945	−1.9324	0.1629
1	M1	1	M2	−0.3458	0.5040	−1.3934	0.7018
2	M1	2	M2	−0.09279	0.8572	−1.1404	0.9548

## Discussion

We report in this manuscript a sensitive and high-throughput assay to study plasma membrane repair. This assay can be used with various adherent cell types such as epithelial cells (Figures 1–4), myocytes (Figure 5), and hepatocytes (our data not shown). Interestingly, we observed that the tested cell types display similar sensitivity to LLO and similar repair efficiencies. This characteristic ensures the versatility of the assay for use with various cell models. Furthermore, this assay could be used to compare the resealing efficiencies of primary cells and cell lines of different origins to document the potential differences in the repair efficiency and/or mechanism between those cells.

Some plate readers are equipped with a microscope objective allowing phase-contrast and fluorescence imaging of cells as their fluorescence intensity is measured. This allows for the determination of cell density and if distinct cell populations co-exist in a given experimental condition. In particular, we noticed that in experimental conditions preventing plasma membrane repair, nearly all cells showed uptake of the PI dye. Conversely, under conditions that allowed for membrane repair, we could observe a population of PI negative cells that had presumably efficiently resealed their plasma membrane (Figure 3B). Therefore, this assay allows refined qualitative and quantitative analysis of cellular subpopulations. Importantly, this assay can be a standalone method to identify or screen for molecules involved in the repair machinery or can be combined with other methods to generate more robust quantitative and qualitative datasets for a comprehensive understanding of plasma membrane repair.

It should be noted that while our assay measured fluorescence intensities at 5 min time intervals for a total of 30 min, the standard read time for a 96-well plate is 25–30 s; therefore, the assay can be adapted to perform kinetics with shorter time intervals if necessary. Alternatively, this assay could be used as an end point assay (Table 3C).

Importantly, the cell density was unaltered in any experimental conditions used in this work. Using distinct cell lines and/or experimental conditions will require ensuring that cells do not detach, which would significantly complicate data interpretation. Thus, we recommend the use of a plate reader that has imaging capability to ensure that cells do not detach. If cell detachment cannot be avoided, a second fluorochrome should be added to estimate cell density. This would allow for correction of the PI intensity based upon the cell density. To damage the plasma membrane, we used the CDC listeriolysin O because our laboratory studies the effects of LLO on mammalian cells. However, other CDCs could be used to replace LLO. Indeed, CDC members are highly homologous and form large transmembrane pores of similar size in cholesterol-rich membranes (Tweten et al., 2015). However, a few CDC members display species specificity, as for example intermedilysin (ILY) produced by *Streptococcus intermedius*, which is specific for human cells (Tweten et al., 2015). Since no systematic comparative study of the membrane repair in response to various CDCs has been reported, there is no information indicating that we should anticipate differences in membrane wounding and or repair in response to different CDC members. One characteristic that distinguishes LLO from all other CDC members is its conformational instability at neutral pH and 37°C. At this temperature and pH, LLO molecules that are not bound to a lipid bilayer become inactive within a few minutes (Schuerch et al., 2005; Vadia et al., 2011). Therefore, LLO purification and manipulation must be performed strictly at 4°C before the toxin is added to mammalian cells at 37°C. It is worth noting that LLO conformational instability is a virtue in this assay because unbound LLO molecules rapidly become inactive, which substantially limits the continuous formation of toxin pores over the duration of the assay and alleviates the requirement to wash the cells after addition of the toxin.

Our current knowledge about the membrane repair machinery is still limited. In particular, it is unclear if different damage conditions elicit various repair mechanisms to reseal the plasma membrane (Andrews et al., 2015; Blazek et al., 2015). Previous studies that analyzed in parallel mechanical- and CDC-pore-induced-wounding concluded that both types of damage triggered a similar repair response (Idone et al., 2008; Corrotte et al., 2013). However, other studies, presented evidence that the size of the wound, for example, affected the mechanism of plasma membrane repair (Jimenez et al., 2015). Therefore, using the CDC-pore-induced membrane damage for the general study of membrane repair is a convenient model, but we cannot rule out that under different damage conditions, there may be other repair mechanisms that are also recruited in response to compromised membrane integrity.

Overall, this assay is a promising tool for the discovery of effectors of the cell repair machinery and for the screening of compounds intended to develop new therapeutic approaches for the treatment of diseases associated with compromised membrane repair capacity.

## Author contributions

SPS designed and performed all of the experimental work, prepared the manuscript and figures. XZ performed the statistical analyses and prepared the manuscript and Tables. JGTL edited the manuscript and prepared the figures. NW provided the C2C12 and H9C9 cells and corresponding cell culture methods as well as prepared the manuscript. SMS designed the assay, prepared the manuscript and figures.

### Conflict of interest statement

The authors declare that the research was conducted in the absence of any commercial or financial relationships that could be construed as a potential conflict of interest.

## Supplementary material

Supplemental Figure 1 Representation of the standard deviations of the Figures 2, 5 data. HeLa cells graphs from Figures 2A,B. C2C12 cells graphs from Figures 5C,D. Data are the average fluorescence intensities expressed in arbitrary unit ± standard deviation of four independent experiments.
