# Supplementary figures and images for: High-Throughput Microplate-Based Assay to Monitor Plasma Membrane Wounding and Repair
Source: Front Cell Infect Microbiol. 2017 Jul 14;7:305. doi: 10.3389/fcimb.2017.00305 (PMC5509797; doi:10.3389/fcimb.2017.00305)

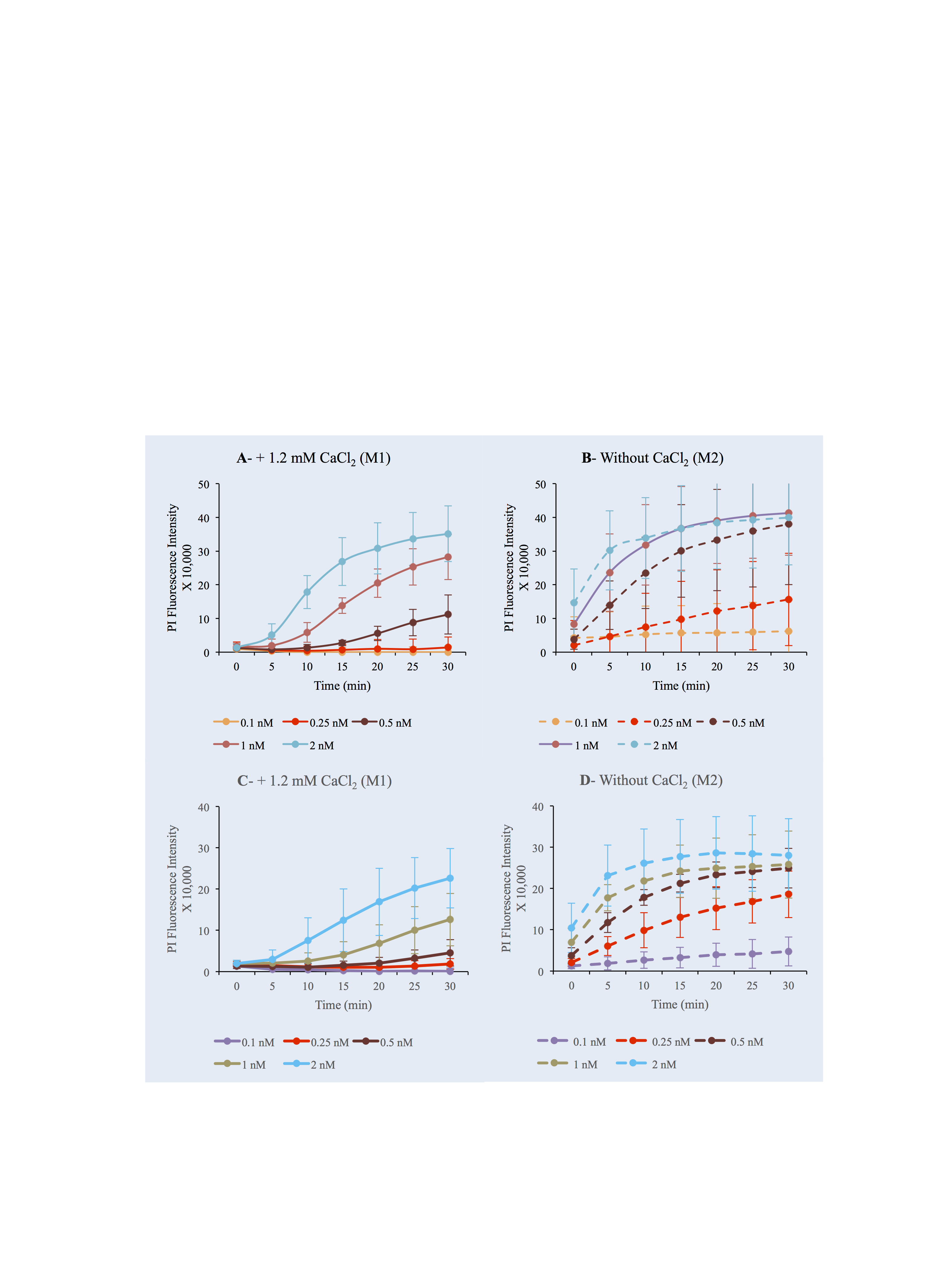

Supplement: Supplemental Figure 1 — Representation of the standard deviations of the Figures 2, 5 data. HeLa cells graphs from Figures 2A,B. C2C12 cells graphs from Figures 5C,D. Data are the average fluorescence intensities expressed in arbitrary unit ± standard deviation of four independent experiments. [file Image1.JPEG]
